# Longitudinal course of temporomandibular joint sounds in Japanese children and adolescents

**DOI:** 10.1186/1746-160X-7-17

**Published:** 2011-10-27

**Authors:** Kengo Torii

**Affiliations:** 1 Department of Prosthodontics, School of Life Dentistry, Nippon Dental University, 1-9-20 Fujimi, Chiyoda-ku, Tokyo, 102-8159, Japan

## Abstract

**Background:**

Many epidemiological studies of temporomandibular disorders (TMDs) in children and adolescents have been performed. However, the results of such studies have varied, and a comprehensive view of the prevalence and severity of symptoms and signs is difficult to obtain. In the present study, temporomandibular joint (TMJ) sounds, which are the most common signs and symptoms of TMD, were observed longitudinally, and the need for treatment was evaluated.

**Methods:**

Seventy individuals in six age groups (5, 6, 7, 8, 9, and 10 years old at the beginning of the observation period) participated in an epidemiological investigation conducted between 1987 and 1992. During each clinical examination, the following parameters were examined: maximum unassisted jaw opening, TMJ clicking, and the coincidence or difference between the midlines of the upper central incisors and the lower central incisors. In addition, the bilateral bite force was recorded, and the numbers of erupted, decayed, and filled teeth were recorded.

**Results:**

Eight individuals dropped out because they moved. No TMJ dysfunction requiring treatment was observed in this series. TMJ clicking was observed in 30 subjects (48%); however, this symptom was temporary in most of subjects (26 subjects; 42%), and only 3 subjects (5%) had persistent clicking (continuing until the end of the observation period). The incidences of clicking were not significantly different among the six groups (x^2 ^= 4.265). Clicking was significantly more common among girls (19 subjects) than among boys (11 subjects; P = 0.042). A significantly lower bite force (17 ± 18 kg) was recorded for the subjects with persistent clicking, compared with that of the other subjects (8- and 9-year-olds; mixed dentition) without persistent clicking (32 ± 17 kg). The persistent clicking began at an age of 11 or 12 years (permanent dentition).

**Conclusion:**

Most of the clicking observed in the children and adolescents was temporary, and no difference in the incidence of clicking was observed among the six age groups. Girls had a significantly higher incidence of clicking than boys.

## Background

Since the end of the 1970s, several epidemiological studies on the signs and symptoms of temporomandibular disorder (TMD) in children and adolescents have been performed [1-9]. However, the frequencies of the most common signs and symptoms varied greatly among these studies. In addition, whether these signs and symptoms were persistent was unclear. Therefore, the present longitudinal study focused on the most common and greater relevant sign of TMD [2,5,8]: temporomandibular joint (TMJ) sounds. Since

TMJ are reportedly related to the occlusal discrepancy between the habitual occlusal position and the bite plate-induced occlusal position [10], the present study also recorded the bite force [11] and the number of decayed and filled teeth in each subject as possible causative factors of the occlusal discrepancy [12].

## Materials and methods

The present epidemiological study was performed during regular oral health examinations performed in the spring and autumn of each year at Ikawa kindergarten, Ikawa elementary school and Ikawa junior high school in Shizuoka city. The study protocol was approved by the department of prosthodontics, Nippon Dental University, because the university did not have an ethical committee at that time. The study was also approved by the local education authority and the parent's council. All the parents of the participants provided their oral informed consent. The participants were divided into six groups: the group 1 consisted of 11 subjects who were 5 years old at the beginning of the study, group 2 consisted of 13 subjects who were 6 years old, group 3 consisted of 13 subjects who were 7 years old, group 4 consisted of 13 subjects who were 8 years old, group 5 consisted of 8 subjects who were 9 years old, and group 6 consisted of 10 subjects aged 10 years old. The examinations were performed twice (in spring and autumn) each year from 1987 to 1992. In 1987 and 1989, only one examination was performed each year. Eight participants dropped out of study because they moved.

### Clinical examinations

All the individuals participating in the study were examined clinically by two experienced dentists according to the specifications for TMD examinations [13]. The following parameters were recorded:

1. Maximum unassisted jaw opening.

2. Deflection on jaw opening.

3. TMJ clicking or crepitations, TMJ locking, TMJ luxation and TMJ pain.

4. Coincidence or difference between the midlines of the upper central incisors and the lower central incisors.

5. Numbers of decayed teeth or filled teeth.

6. Number of erupted teeth.

In addition, another dentist measured the bite force at the second deciduous molars in the primary dentition or the first molars in mixed or permanent dentitions using an occlusal force meter (MPM 3000, NIHON KOHDEN, Tokyo, Japan). Three measurements were performed on each side after asking the subject to bite as strongly as possible, and the mean value for each side was totaled and regarded as the bilateral bite force [11].

### Statistical analysis

A chi-square test was used to analyze the distributions of the variables in different groups of subjects on a nominal scale and the Fisher exact test was also used on a nominal scale. The statistical differences between mean values were evaluated using a t-test (including the Cochran-Cox method).

## Results

None of the subjects with TMJ dysfunction required treatment in this series. The distribution of the incidence of TMJ clicking is shown in Table 1. Clicking was observed in 30 subjects (48%); however, this symptom was temporary (26 subjects; 42%), and only 3 subjects (5%) had persistent clicking (continuing until the end of the observation period). The incidences of clicking were not significantly different among the six groups (Table 2). Clicking was significantly more common among girls than among boys (Table 3). The midline difference between the upper and lower central incisors was not correlated with clicking (Table 4). Six subjects had clicking for more than two years, and the maximum unassisted opening, maximum bite force, and the number of decayed and filled teeth among these subjects were compared with these parameters in other subjects belonging to the same age group. No statistically significant differences in the maximum opening were found between subjects with and those without persistent clicking (Table 5). Regarding the maximum bite force, only two subjects (No. 31 and 46) with persistent clicking had a significantly lower maximum bite force than the other subjects during the early period of observation (their ages were 8 and 9 years old, respectively; mixed dentition; see Table 6). No significant differences in the number of decayed, filled, and the total number of decayed and/or filled teeth were found between subjects with and those without persistent clicking (Table 7).

**Table 1:** Incidences of TMJ clicking among six age groups

Group	**No**.	1987	1988		1989		1990		1991		1992	
	2						□	□				
	6		□				□				□	
G1	7							□		□		
	10	□						□				

	12					□	□	□	□	□	□	
	13							□				
	18	□	□	□		□	□	□				
G2	21						□					
	23						□			□		
	24						□					

	25						□					
	27						□	□				
	30		□							□		
G3	31	□			□		■	□	□	□	□	
	32		□					□				
	33	□										
	34	□										

												
	37	□										
	38											□
	39			□			□	□				
G4	40						□					
	42							□			□	
	46	□					□	□	□	□	□	□
	47	□		□								

G5	49							□				

	59		□	□		□	□	□	□	□	■	■
	60	□		□				□	□	□	■	■
G6	65	□	□	□		□						
	67											□
	68					□						
												

G1: group of subjects aged 5 years old at beginning of observation; G2: group of subjects aged 6 years old; G3: group of subjects aged 7 years old; G4: group of subjects aged 8 years old; G5: group of subjects aged 9byears old and G6: group of subjects aged 10 years old. The examinations were performed twice each year except in 1987 and 1989 (only one examination was performed in each these years). No.: Subject number; □: Clicking; ■: Crepitation.

**Table 2:** Contingency table for the number of subjects with and those without TMJ clicking

Group	with clicking	without clicking	total
G1	5	4	9
G2	6	6	12
G3	3	7	10
G4	6	7	13
G5	4	1	5
G6	8	5	13

X^2 ^= 4.265 < X^2^(P = 0.5): not significant.

**Table 3:** Contingency table for male and female subjects with or without TMJ clicking

Gender	with clicking	without clicking	total
Male	11	19	32
Female	19	12	30

P = 0.042 (The relationship between girls and clicking was significant when evaluated using the Fisher exact test).

**Table 4:** Contingency table for number of subjects with and those without TMJ clicking related to difference or coincidence between the midlines of the upper central incisors and the lower central incisors

Midline discrepancy	with clicking	without clicking	total
No discrepancy	16	16	32
Discrepancy	18	12	30

P = 0.149: not significant.

**Table 5:** Unassisted maximum opening (mm) in subjects with and those without persistent TMJ clicking (continuing for more than two years)

Subjects	1988	1989	1990	1991	1992
No. 12 and 18	41.0 ± 0	41.5 ± 2.1	45.0 ± 4.2	50.0 ± 1.4	45.0 ± 0
Other subjects	42.4 ± 2.8	44.7 ± 4.4	45.6 ± 3.4	45.8 ± 3.2	45.4 ± 3.2
No. 31 and 46	45.5 ± 6.4	48.0 ± 4.2	47.0 ± 1.4	44.5 ± 0.7	47.0 ± 4.2
Other subjects	42.5 ± 4.2	45.5 ± 1.0	46.1 ± 1.0	47.0 ± 3.0	56.9 ± 5.5
No. 59 and 60	49.7 ± 3.2	53.0 ± 5.6	57.5 ± 4.9	60.5 ± 7.8	62.0 ± 5.7
Other subjects	45.0 ± 4.8	47.7 ± 4.0	47.8 ± 3.9	49.5 ± 3.9	50.4 ± 3.5

**Table 6:** Maximum bite force (kg) of subjects with and those without persistent TMJ clicking (continuing for more than two years)

Subjects	1988	1989	1990	1991	1992
No. 12 and 18	29.5 ± 2.1	39.5 ± 2.1	39.5 ± 12.0	72.0 ± 19.8	50.5 ± 12.0
Other subjects	30.4 ± 12.5	36.8 ± 17.7	47.1 ± 20.6	46.6 ± 20.0	50.7 ± 27.1
No. 31 and 46	17.0 ± 18.4 †	27.0 ± 21.2	25.5 ± 26.2	17.0 ± 14.1	38.0 ± 31.1
Other subjects	31.5 ± 17.0	40.0 ± 23.2	50.0 ± 27.1	44.0 ± 22.5	52.8 ± 17.1
No. 59 and 60	29.7 ± 10.0	50.7 ± 10.0	46.5 ± 13.4	58.0 ± 0	49.0 ± 11.3
Other subjects	43.8 ± 8.6	52.6 ± 17.7	56.6 ± 22.0	62.8 ± 31.4	75.3 ± 31.3

†P < 0.005: significant

**Table 7:** Number of filled (T1), decayed (T2) and total number of filled and/or decayed (T1 + T2) of subjects with or without persistent TMJ clicking (continuing for more than two years)

Subjects	1988	1989	1990	1991	1992
No. 12 and 18					
T1	4.0 ± 0	n.r.	3.5 ± 2.1	8.0 ± 1.4	1.0 ± 1.4
T2	7.5 ± 0.7	n.r.	7.0 ± 2.8	2.5 ± 3.5	1.0 ± 1.4
T1 + T2	11.5 ± 0.7	n.r.	10.5 ± 4.9	10.5 ± 4.9	2.0 ± 0
Other subjects					
T1	2.5 ± 2.8	n.r.	5.6 ± 3.5	7.3 ± 4.0	3.0 ± 2.1
T2	3.2 ± 4.1	n.r.	2.6 ± 3.4	1.0 ± 1.0	0.2 ± 0.5
T1 + T2	5.7 ± 5.2	n.r.	8.5 ± 5.2	8.3 ± 4.5	3.3 ± 1.8
No. 31 and 46					
T1	3.0 ± 4.2	n.r.	0	7.5 ± 2.1	1.0 ± 1.4
T2	7.5 ± 7.8	n.r.	6.0 ± 8.5	3.5 ± 4.9	3.5 ± 4.9
T1 and T2	10.5 ± 3.5	n.r.	6.0 ± 8.5	11.0 ± 2.8	4.5 ± 3.5
Other subjects					
T1	3.6 ± 2.9	n.r.	5.5 ± 3.0	6.6 ± 3.5	2.6 ± 2.1
T2	3.6 ± 3.4	n.r.	1.7 ± 2.3	1.0 ± 1.2	1.2 ± 1.9
T1 + T2	7.4 ± 3.9	n.r.	7.2 ± 3.6	7.7 ± 3.6	3.8 ± 2.7
No. 59 and 60					
T1	2.0 ± 0	n.r.	2.5 ± 2.1	3.0 ± 1.4	5.5 ± 0.7
T2	2.0 ± 0	n.r.	1.5 ± 2.1	2.5 ± 2.1	0.5 ± 0.7
T1 + T2	4.0 ± 0	n.r.	4.0 ± 0	5.5 ± 0.7	6.0 ± 0
Other subjects					
T1	2.2 ± 1.8	n.r.	3.4 ± 1.9	3.4 ± 1.1	3.8 ± 1.6
T2	2.7 ± 2.8	n.r.	1.1 ± 1.6	1.1 ± 1.4	0.7 ± 0.8
T1 + T2	5.0 ±	n.r.	4.5 ± 2.0	5.5 ± 0.7	4.6 ± 1.9

n.r.: not registered

## Discussion

Kӧhler et al. reported in their cross-sectional epidemiological study covering two decades that the incidences of TMJ clicking and crepitation in 10-year-old children were 14% in 1983, 3% in 1993, and 5% in 2003. Widmalm et al. [4] reported that the percentage of TMJ sounds was 47.8% among 4-6-year-old children. Magnusson et al. [3] reported in their longitudinal epidemiological study that the incidence of TMJ sounds changed from 5% among 7-year-old subjects to 30% among 11-year-old subjects. Recently, Tecco et al. reported that the percentages of TMJ sounds were 1.75% among subjects aged 5-11 years old and 8.21% among subjects aged 12-15 years old. In the present study, the overall incidence of TMJ clicking was 48.3%, but when calculated on a yearly basis, the incidences were 15% in 1987, 11% in 1988, 13% in 1989, 19% in 1990, 11% in1991 and 11% in 1992. Thus, the incidence fluctuated over time. In the present study, most of the TMJ clicking was temporary, but persistent clicking was observed in 3 subjects. The persistent clicking began when the subjects were 11 or 12 years old, and the clicking ultimately changed to crepitation. Persistent clicking is thought to be a more important symptom than temporary clicking, since persistent clicking or crepitation can cause TMJ locking, resulting in a need for treatment. In the studies reported by Widmalm et al. [4] and Tecco et al. [6], the difference in incidence according to sex was not significant; in the present study, however, the incidence was higher among girls than among boys. Magnusson et al. [7] reported that the incidence of one or more subjective symptoms of TMJ sounds, jaw fatigue, difficulties in mouth opening, and pain during chewing was higher among women than among men (P < 0.05). The higher incidence among girls in the present study may reflect the higher tendency of TMD symptoms to appear in women. Magnusson et al. [7] reported that a positive correlation was found between subjective TMJ sound and one or more subjective symptoms of TMD. Kӧnӧnen and Nystrӧm [8] reported that TMJ sounds were the most frequent findings and increased with age, but only a few patients consistently reported clicking sounds or had them recorded in their longitudinal study.

In subjects less than 10 years of age, the dentition is generally a mixture of deciduous and permanent teeth. During the mixed dentition stage, numerous occlusal interferences are present, and the muscles must repeatedly learn new patterns of mandibular closure to avoid interfering or decayed teeth. The pathway of centric relation (muscular centric position), having only been established in the central nervous system for a short time, is not as entrenched as it is in adults. No repeatedly used position of occlusal contact that does not coincide with the centric relation is ever acquired by chance.

Consequently, a discrepancy between the habitual occlusal position and the muscular centric position (bite plate-induced occlusal position) emerges, resulting in temporary clicking. In the present study, as no relationships were observed between persistent clicking and the numbers of decayed, filled or total decayed and/or filled teeth, the avoidance of decayed or filled teeth was not thought to be a cause of the occlusal discrepancy. These results suggest that persistent clicking was not caused by iatrogenic factors. Generally, teeth that erupt in a malposition that does not coincide with the centric relation may have been repositioned into the correct position coinciding with the centric relation by muscular force, that is the bite force. Under conditions in which an occlusal discrepancy exists until the tooth is repositioned into the correct position, temporary clicking may be produced. However, if the bite force is small before the completion of the permanent dentition, the malposition of the occlusion will be maintained. Once the permanent dentition stage has been reached, usually at an age of 10-12 years, the muscles frequently adopt an occlusal position that does not coincide with the centric relation. This new position of occlusal contact usually begins as an expedient process to avoid interferences, providing better function than the centric relation provides at that moment. The continued presence of the occlusal discrepancy causes the new reflex pattern of the pathway to be used so repeatedly that the new position of the mandible may resemble the centric relation. This acquired position can be regarded as the usual or habitual occlusal position [12]. In the present study, subjects No. 31 and 46 had a significantly smaller bite force at the ages 8 and 9 years old, respectively (mixed dentition); thus, the occlusal discrepancy may have appeared as a result of the small bite force, and this discrepancy may have been maintained once the permanent dentition was achieved, resulting in the persistent clicking. Although no data on the occlusal force at the ages of 8 or 9 years were available for subjects No. 59 and 60, the occlusal forces of these two subjects tended to be smaller than those of the others at later stage (difference not significant; see Table 6). Therefore, the persistent clicking in these subjects was thought to have been caused by an occlusal discrepancy that emerged as a result of a small bite force during the mixed-dentition stage. For individuals with persistent clicking, the examiner in charge of routine dental and oral health examinations should tell the subject to visit a dental clinic if TMJ locking or other TMD symptoms should appear.

## Conclusions

Although the population size in the present study was relatively small, the following conclusions were obtained:

1. Among 5-15-year-old children and adolescents, most cases of TMJ clicking were temporary, and no differences in the incidence of clicking were observed among the six age groups.

2. Girls had a significantly higher incidence of clicking than boys.

3. A few subjects had persistent clicking, beginning at 11 or 12 years of age once the permanent dentition had been achieved.

## Competing interests

The author declares that they have no competing interests.

## Authors' contributions

KT conceived the study, participated in the study's design, and performed clinical examination and statistical analysis.

## Author details

Visiting professor, School of Life Dentistry, Nippon Dental University, Tokyo, 1-9-20 Fujimi, Chiyoda-ku, Tokyo, 102-8159 Japan
